# Examining the role of reinforcing activities and time horizon in recovery: Commentary on Bickel, Witkiewitz, Athamneh, Kuhlemeier—“Recovery from alcohol use disorder: Reinforcer pathology theory, measurement, and methods”

**DOI:** 10.1111/acer.15466

**Published:** 2024-10-15

**Authors:** James R. McKay

**Affiliations:** ^1^ Department of Psychiatry University of Pennsylvania, and Department of Veterans Affairs Medical Center Philadelphia Pennsylvania USA

The article by Bickel et al. (2024) makes a case for a new theory of recovery from alcohol and drug use disorders, referred to as “Reinforcer Pathology Theory,” or RP. Two of the central assertions of RP are that recovery is aided by (1) greater involvement in reinforcing alcohol‐ or drug‐free activities, and (2) extending one's temporal window, so less delay discounting occurs (Bickel et al., 2017). The authors provide a helpful and concise review of research findings that support the importance of these two factors in explaining how individuals reduce the use of alcohol and other drugs. However, they note that almost all the research has been correlational and focused specifically on substance use rather than recovery, which according to a new National Institute on Alcohol and Alcoholism (NIAAA) definition also includes improvements in psychosocial functioning and quality of life (Hagman et al., 2022). The article looks at how RP theory may also apply to the concept of recovery, presents new data that is said to support the theory, and describes a new NIAAA‐funded study that examines predictors and correlates of recovery over 12 years with a novel, accelerated longitudinal design.

The theoretical basis behind RP is convincing. The theory matches up well with research findings on delay discounting and clinical observations regarding the importance to recovery of engagement in meaningful, rewarding activities, as well as the problems posed by too much of a continued preference for immediate rewards. There seems to be no obvious reason why prior research findings regarding delayed discounting focused on abstinence would not apply to the broader concept of recovery as well. However, given that many existing treatments devote at least some attention to increasing time spent in rewarding activities and addressing impulsivity and preference for immediate rewards (e.g., cognitive behavioral therapy [CBT], community reinforcement approach, and Alcoholics Anonymous [AA]), I am not sure that RP should be considered a new theory. Rather, it seems more a repackaging of ideas about important factors in addiction and recovery that have been with us for a while now, but perhaps have not been put together in this manner before.

To provide support for RP, the authors present new analyses from Project MATCH (Project MATCH Research Group, 1997), which demonstrate that more alcohol‐free activities and higher reinforcing value of alcohol‐free activities assessed 6 months after the end of treatment are associated with a greater likelihood of being in recovery about 2 years later. They also present a new 5‐year study that makes use of a novel, accelerated longitudinal design to be able to examine predictors of recovery over a 12‐year period. These sections of the article are fascinating, and I eagerly look forward to seeing the results of the new study. To their credit, the authors are also clear about the limitations of these new Project MATCH analyses and of the new study. These include an older data set, limited numbers of women and people of color, correlational analyses, and lack of more frequent assessments of rewarding activities (Project MATCH); and not knowing to what extent the sample will be broadly representative of individuals in recovery, the need for participants to have internet access, and reliance on remotely administered assessments and surveys without biological confirmation of alcohol use (new NIAAA R01). Although the authors do not list this as a limitation, the design of the new accelerated longitudinal study really only allows for correlational analyses as there are no experimental manipulations, if I'm understanding it correctly.

In my heart of hearts, I strongly believe that we need to devote more attention and effort toward finding ways to increase the time that individuals seeking recovery spend in rewarding activities, which motivated me to write a position piece on this a few years ago (McKay, 2017). Our current evidence‐based treatments are more focused on getting rid of problematic behaviors and cognitions than on increasing activities that make life more enjoyable and meaningful, which may render them less compelling to some of those who could conceivably benefit from them. And it is hard to argue with the notion that lengthening the time horizon to reduce delay discounting helps with therapeutic efforts to shift patients' focus toward lower intensity, less predictable rewarding experiences that require a great deal of effort, such as enhancing relationships, developing hobbies, and following other passions. However, I'm concerned that correlational designs, even when innovative, will not truly nail down whether the relation between changes in rewarding activities and delay discounting and subsequent changes in substance use/recovery status is in fact causal. To do that, we need experimental designs. One such design might randomize participants to two or more levels of rewarding activities, to test whether being in the condition that provided more rewarding activities produced higher rates of recovery. This approach, however, would not be feasible with delay discounting, as it is not really possible to randomize individuals to do more versus less of this.

A second approach would instead randomize participants to treatment as usual or to one or more interventions designed to increase the frequency of rewarding activities and/or reduce delay discounting. There is already a very successful demonstration of the power of this sort of design in alcohol use disorder treatment research. For years, it was difficult if not impossible to conduct a random assignment, controlled study of AA‐oriented treatments. However, the investigators who conducted NIAAA's Project MATCH came up with the idea to develop and test an intervention focused specifically on getting participants to go to AA meetings and become more involved in the program (e.g., talking at meetings, getting a sponsor, working the steps, and getting together with others in the program outside of the meetings). The intervention was referred to as Twelve‐Step Facilitation (TSF) (Nowinski et al., 1992). Analysis of the Project MATCH data indicated that it was at least as effective in reducing drinking frequency as CBT or MET and was superior when the outcome was total abstinence (Project MATCH Research Group, 1997, 1998). Mediation analyses indicated that TSF resulted in higher levels of AA affiliation and this led to better outcomes in TSF for those with social supports that encouraged drinking (Longabaugh et al., 2001). Further analyses examined potential mediators of the superior outcomes on absolute abstinence for TSF as compared to MET and CBT. TST produced higher scores on commitment to AA practices than either MET or CBT, and commitment to AA practices mediated, or accounted for, the observed differential outcomes (Longabaugh et al., 2005). Therefore, these analyses demonstrated that TSF accomplished its goal of getting patients more involved in AA and that this is what accounted for its beneficial effects.

Mark Litt and colleagues at the University of Connecticut broadened the goal of TSF to also include other rewarding activities outside of mutual help. This new intervention, referred to as Network Support (NS), was evaluated in two RCTs in which it was compared with case management (Litt et al., 2007, 2009) and CBT (Litt et al., 2016). In both studies, NS produced better alcohol use outcomes than the comparator conditions, as well as higher rates of participation in AA and a greater proportion of nondrinkers in the social network. Further analyses in both studies indicated that the positive effect of NS on alcohol use outcomes was mediated by pre‐ to posttreatment increases in self‐efficacy and social network variables and attendance at AA.

It should be noted that getting people to participate more in AA may be an easier lift than increasing time spent in other rewarding activities. Individuals may need a fair amount of help in figuring out the activities or experiences that will be rewarding for them, these activities and experiences must be readily available, and the resources to access them need to be obtained somehow. The field of leisure science has devoted considerable effort to developing and testing interventions designed to increase time spent in these sorts of activities (Fancourt et al., 2021; Iwasaki, 2017). This may be a good source of ideas and potential collaborators for addiction treatment researchers interested in developing interventions to increase time spent in rewarding activities.

The authors mention only one approach to extending temporal windows, episodic future thinking. Good therapy for externalizing adolescents and young adults usually includes some efforts to increase willingness to work for more distal goals and rewards, to counteract the overvaluing of immediate rewards. However, this is slow work that requires skilled psychotherapists and a lot of sessions. There clearly is a need for more efficient, low‐cost interventions that reduce delay discounting. In the VA's nationwide rollout of CM (DePhilippis et al., 2023), we have observed anecdotally that many Vets decide to hang on to their rewards to save up for more expensive items, rather than cash them in at every session. Although this likely reflects pretreatment individual differences in delay discounting, it could also be an indication that the Veterans' time horizons have been expanded. We unfortunately do not have the data in VA to test whether saving up rewards predicts better outcomes. Three other studies have examined this issue and have come to differing conclusions (Fletcher et al., 2014; Krishnamurti et al., 2020; Murtaugh et al., 2013). But it does raise intriguing questions about whether simply knowing that you've earned a reward makes it easier to “save” it for a later day, thereby stretching your time horizon.

## CONFLICT OF INTEREST STATEMENT

The author reports no conflicts of interest.
